# Cost-effectiveness of Dapagliflozin for the Treatment of Heart Failure With Reduced Ejection Fraction

**DOI:** 10.1001/jamanetworkopen.2021.14501

**Published:** 2021-07-27

**Authors:** Nicolas Isaza, Paola Calvachi, Inbar Raber, Chia-Liang Liu, Brandon K. Bellows, Inmaculada Hernandez, Changyu Shen, Michael C. Gavin, A. Reshad Garan, Dhruv S. Kazi

**Affiliations:** 1 Department of Internal Medicine, Beth Israel Deaconess Medical Center, Boston, Massachusetts; 2 Harvard Medical School, Boston, Massachusetts; 3 Division of Cardiology, Beth Israel Deaconess Medical Center, Boston, Massachusetts; 4 Richard A. and Susan F. Smith Center for Outcomes Research in Cardiology, Boston, Massachusetts; 5 Harvard T.H. Chan School of Public Health, Boston, Massachusetts; 6 Division of General Medicine, Columbia University Department of Medicine, New York City, New York; 7 School of Pharmacy and Pharmaceutical Science, University of California, San Diego

## Abstract

**Importance:**

Heart failure with reduced ejection fraction produces substantial morbidity, mortality, and health care costs. Dapagliflozin is the first sodium-glucose cotransporter 2 inhibitor approved for the treatment of heart failure with reduced ejection fraction.

**Objective:**

To examine the cost-effectiveness of adding dapagliflozin to guideline-directed medical therapy for heart failure with reduced ejection fraction in patients with or without diabetes.

**Design, Setting, and Participants:**

This economic evaluation developed and used a Markov cohort model that compared dapagliflozin and guideline-directed medical therapy with guideline-directed medical therapy alone in a hypothetical cohort of US adults with similar clinical characteristics as participants of the Dapagliflozin in Patients with Heart Failure and Reduced Ejection Fraction (DAPA-HF) trial. Dapagliflozin was assumed to cost $4192 annually. Nonparametric modeling was used to estimate long-term survival. Deterministic and probabilistic sensitivity analyses examined the impact of parameter uncertainty. Data were analyzed between September 2019 and January 2021.

**Main Outcomes and Measures:**

Lifetime incremental cost-effectiveness ratio in 2020 US dollars per quality-adjusted life-year (QALY) gained.

**Results:**

The simulated cohort had a starting age of 66 years, and 41.8% had diabetes at baseline. Median (interquartile range) survival in the guideline-directed medical therapy arm was 6.8 (3.5-11.3) years. Dapagliflozin was projected to add 0.63 (95% uncertainty interval [UI], 0.25-1.15) QALYs at an incremental lifetime cost of $42 800 (95% UI, $37 100-$50 300), for an incremental cost-effectiveness ratio of $68 300 per QALY gained (95% UI, $54 600-$117 600 per QALY gained; cost-effective in 94% of probabilistic simulations at a threshold of $100 000 per QALY gained). Findings were similar in individuals with or without diabetes but were sensitive to drug cost.

**Conclusions and Relevance:**

In this study, adding dapagliflozin to guideline-directed medical therapy was projected to improve long-term clinical outcomes in patients with heart failure with reduced ejection fraction and be cost-effective at current US prices. Scalable strategies for improving uptake of dapagliflozin may improve long-term outcomes in patients with heart failure with reduced ejection fraction.

## Key Points

**Question:**

Is the addition of dapagliflozin to guideline-directed medical therapy cost-effective for the treatment of heart failure with reduced ejection fraction?

**Findings:**

In this economic evaluation of a simulated cohort of US adults with heart failure, adding dapagliflozin to guideline-directed medical therapy was projected to prolong survival by 0.63 quality-adjusted life-years while increasing lifetime costs by $42 800, producing an incremental cost-effectiveness ratio of $68 300 per quality-adjusted life-year. Results in individuals with and without diabetes were similar.

**Meaning:**

These results suggest that widespread uptake of dapagliflozin for the treatment of heart failure with reduced ejection fraction has the potential to improve long-term clinical outcomes and is likely to meet conventional cost-effectiveness thresholds.

## Teaser

This economic evaluation examined the cost-effectiveness of adding dapagliflozin to guideline-directed medical therapy for heart failure with reduced ejection fraction in a simulated cohort of patients with or without diabetes.

## Introduction

Sodium glucose cotransporter 2 (SGLT2) inhibitors were developed for the treatment of diabetes but were incidentally noted to improve cardiovascular outcomes.^1,2,3,4,5^ In the Dapagliflozin in Patients with Heart Failure and Reduced Ejection Fraction (DAPA-HF) trial, adding dapagliflozin to guideline-directed medical therapy (GDMT) for heart failure with reduced ejection fraction (HFrEF) reduced the risk of cardiovascular death or heart failure hospitalization by 26% compared with GDMT alone, regardless of the presence or absence of diabetes.^4^ On May 5, 2020, dapagliflozin became the first SGLT2 inhibitor to receive approval from the US Food and Drug Administration (FDA) for the treatment of HFrEF, including among patients without diabetes.^6^

The arrival of a new class of therapeutics for HFrEF is a welcome development, as heart failure remains a prevalent disease that produces substantial morbidity and mortality and generates enormous health care costs. As heart failure is a leading cause of hospitalization in the US, dapagliflozin may improve health outcomes and reduce health care costs by averting heart failure hospitalizations downstream, but widespread uptake at a list price of $6188 per year of treatment could produce a large increase in pharmaceutical expenditures. A systematic cost-effectiveness evaluation from a US health care sector perspective would quantify the trade-off between increased health care costs and improved health outcomes and therefore help inform pricing and adoption strategies. This is particularly important for SGLT2 inhibitors because diabetes therapies have historically seen large manufacturer discounts, suggesting that actual prices faced by payers may be highly variable and subject to negotiation with manufacturers.^7^ We therefore evaluated the cost-effectiveness of adding dapagliflozin to GDMT for the management of HFrEF in patients with or without diabetes at baseline from a US health care sector perspective and a lifetime analytic horizon.

## Methods

### Model Overview

We developed a Markov cohort model to compare 2 treatment strategies among patients with HFrEF: (1) GDMT, comprised of an angiotensin converting enzyme inhibitor, an angiotensin-receptor blocker, or an angiotensin receptor neprilysin inhibitor, in addition to a β-blocker and a mineralocorticoid receptor antagonist^8,9^; and (2) dapagliflozin (10 mg once daily) added to GDMT. The analysis adopted a US health care sector perspective and a lifetime analytic horizon. Future costs and outcomes were discounted at 3% annually.^10^ We adhered to the recommendations of the Second Panel on Cost-Effectiveness in Health and Medicine to the extent feasible.^10^ Given that the study relied on publicly available data sets, this was deemed to not be human participants research and institutional review board approval was not required per the Common Rule. This study was conducted independently from the commercial sponsor of the DAPA-HF trial.

Model creation and analyses were performed using TreeAge Pro Healthcare 2020 (TreeAge Software), and Microsoft Excel version 16 (Microsoft Corporation). Additional modeling details are provided in eMethods in the Supplement.

### Simulated Population

**Figure 1.**
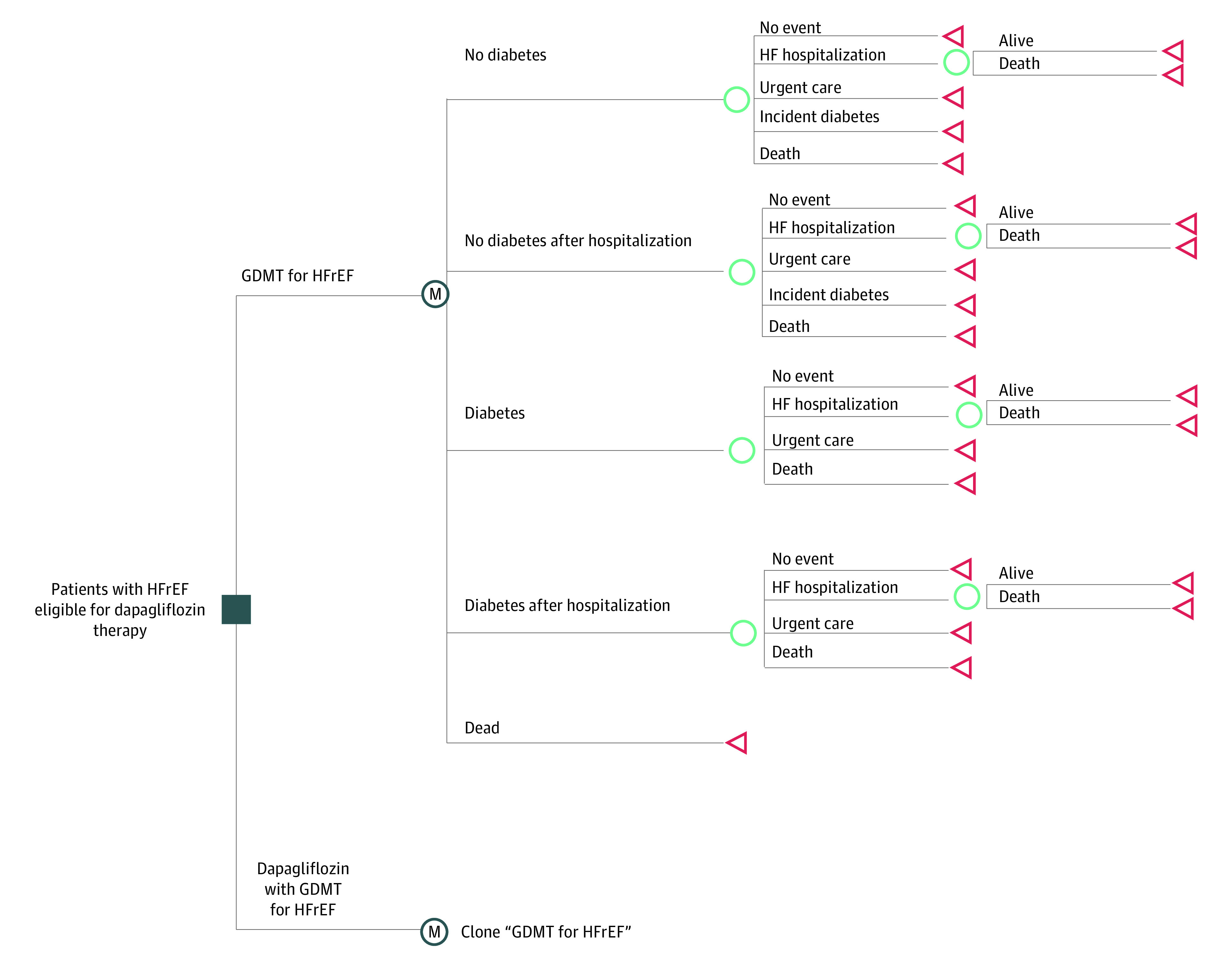
Model Structure The Markov cohort model used in this study simulated a hypothetical cohort of patients with heart failure with reduced ejection fraction (HFrEF) with clinical characteristics similar to the participants of the Dapagliflozin in Patients with Heart Failure and Reduced Ejection Fraction trial. The model compared guideline-directed medical therapy (GDMT) in the control arm with dapagliflozin added to GDMT in the intervention arm. In monthly cycles, patients could continue to live with HF, experience an urgent HF visit, experience a HF hospitalization (with or without a readmission in the first 30 days after the index hospitalization), develop incident diabetes, or die from any cause.

We simulated a hypothetical cohort with characteristics similar to the participants of the DAPA-HF trial, which was a phase 3, placebo-controlled, double-masked, randomized trial that enrolled patients with HFrEF who had New York Heart Association class II, III, or IV symptoms and a left ventricular ejection fraction of 40% or less. Patients with an estimated glomerular filtration rate less than 30 mL/min/1.73 m^2^ of body surface were excluded. The cohort was stratified by baseline diabetes status to capture the heterogeneity of outcomes by diabetes status in the control arm of the DAPA-HF trial (Figure 1).^11^

### Model Inputs

#### Transitional Probabilities

Key model inputs were derived from published primary and secondary analyses of the DAPA-HF, publicly available US regulatory review documents, published clinical and epidemiologic studies, national health care claims data, and the Medical Expenditure Panel Survey (Table 1).^4,11,12,17,18,19,21,22,23,24,25,26^ In monthly cycles, patients could experience a fatal or nonfatal heart failure hospitalization, a heart failure–related urgent care visit, or death from any cause. Patients who were discharged after a heart failure hospitalization in 1 cycle were at increased risk of readmission during the following cycle.^12,13^ Patients with diabetes experienced higher rates of heart failure hospitalization and all-cause mortality than patients without diabetes at baseline. Patients without diabetes at baseline could develop diabetes during follow-up (with the attendant increase in risk of adverse events).^26^

**Table 1.** Input Parameters

Parameter	Base-case value (range in sensitivity analyses)	Distribution for probabilistic analyses	Source
**Transitional probabilities for the GDMT (control) arm**			
Rate of HF hospitalizations, per person year			Petrie et al,^11^ 2020
No diabetes	0.080 (0.071 to 0.089)	β	
Diabetes	0.122 (0.111 to 0.133)	β	
Proportion of HF hospitalizations that are fatal	0.0954 (0.035 to 0.105)	β	Wadhera et al,^12^ 2018
Probability of 30-d readmission after a HF hospitalization	0.20	β	Wadhera et al,^12^ 2018
Proportion of HF-specific readmissions	0.37	β	Krumholz,^13^ 2013
Rate of urgent HF visits, per person year			Petrie et al,^11^ 2020
No diabetes	0.006 (0.003 to 0.009)	β	
Diabetes	0.007 (0.004 to 0.010)	β	
Rate of incident diabetes, per person year	0.037 (0.030 to 0.044)	β	Inzucchi et al,^14^ 2020
All-cause mortality in first 24 mo, per person year^a^			Petrie et al,^11^ 2020
No diabetes	0.078 (0.069 to 0.087)	β	
Diabetes	0.117 (0.106 to 0.128)	β	
Death from any cause (beyond 24 mo)	Ratio for all-cause mortality comparing the control arm to the US general population (see eMethods in the Supplement)		
**Effectiveness of dapagliflozin (intervention arm)**			
HR for HF hospitalizations, compared with GDMT			Petrie et al,^11^ 2020
No diabetes	0.63 (0.48 to 0.81)	Log normal	
Diabetes	0.76 (0.61 to 0.95)	Log normal	
HR for urgent HF visits, compared with GDMT			Petrie et al,^11^ 2020
No diabetes	0.25 (0.07 to 0.89)	Log normal	
Diabetes	0.62 (0.24 to 1.59)	Log normal	
HR for death from any cause compared with GDMT (first 24 mo)			Petrie et al,^11^ 2020
No diabetes	0.88 (0.70 to 1.12)	Log normal	
Diabetes	0.78 (0.63 to 0.97)	Log normal	
HR for incident diabetes compared with GDMT	0.68 (0.50 to 0.94)	Log normal	Inzucchi et al,^14^ 2020
**Costs**			
Dapagliflozin therapy, $/y	4192 (953 to 6188)	Log normal	Base case: FSS-Big 4; lower bound: heavily discounted price; upper bound: wholesale acquisition (all prices estimated August 2020)^15,16^
Background health care costs, $/y			HCUP^17^; Peterson et al,^18^ 2015; Kazi et al,^19^ 2020
No diabetes			
Age, y			
<75	20 629 (16 503 to 24 755)	Log normal	
75-85	22 512 (18 010 to 27 015)	Log normal	
>85	30 811 (24 648 to 36 973)	Log normal	
Diabetes			
Age, y			
<75	28 923 (23 139 to 34 708)	Log normal	
75-85	26 430 (21 144 to 31 716)	Log normal	
>85	34 249 (27 400 to 41 099)	Log normal	
HF hospitalization costs, $	11 827 (8899 to 15 591)	Log normal	Medicare Provider Utilization and Payment Data 2017^20^
Urgent HF visit cost, $	807 (646 to 968)	Log normal	Charges for services provided during an urgent care visit and a cost-center–specific charge-to-payment ratio
**Quality of life**			
Baseline KCCQ-OSS in the GDMT (control) arm	68.6 (68.1 to 69.1)	Normal	Kosiborod et al,^21^ 2020
Baseline KCCQ-OSS in the dapagliflozin (intervention) arm	68.4 (68.1 to 69.1)	Normal	Kosiborod et al,^21^ 2020
KCCQ-OSS in the GDMT (control) arm at 8 mo	72.7 (72.0 to 73.2)	Normal	Kosiborod et al,^21^ 2020
KCCQ-OSS in the dapagliflozin (intervention) arm at 8 mo	75.0 (74.4 to 75.4)	Normal	Kosiborod et al,^21^ 2020
Quality-of-life penalty applied for diagnosis of diabetes	−0.0351 (−0.0350 to −0.0352)	Normal	Sullivan et al,^22^ 2006
Quality-of-life penalty applied for HF hospitalization	−0.0066 (−0.0135 to 0)	Normal	Jaagosild et al,^23^ 1998
Quality-of-life penalty applied for urgent HF visit	−0.0045 (−0.009 to 0)	Normal	Jaagosild et al,^23^ 1998

Abbreviations: FSS, Federal Supply Schedule; GDMT, guideline-directed medical therapy; HCUP, Healthcare Costs and Utilization Project; HF, heart failure; HR, hazard ratio; KCCQ-OSS, Kansas City Cardiomyopathy Questionnaire–Overall Summary Score.

^a^ Ratio for all-cause mortality comparing the control arm with the US general population beyond 24 mo available in the eMethods in the Supplement.

The model was calibrated to reproduce rates of 18-month survival, urgent heart failure visits, and HFrEF hospitalizations observed in the DAPA-HF trial. Therefore, hospitalizations for HFrEF exacerbations were more frequent in patients with diabetes compared with patients without diabetes, but rates of urgent care visits for heart failure were similar in the 2 groups (Table 1).^11^

#### Effectiveness of Dapagliflozin

We assumed that the use of dapagliflozin would reduce HFrEF hospitalization, urgent care visits, and all-cause mortality as observed in the DAPA-HF trial (with the effect size stratified by patients’ diabetes status) (Table 1).^11^ The base case assumed that the effectiveness of dapagliflozin would be sustained over the lifetime of the patients, but a sensitivity analysis assumed a linear decline in effectiveness of dapagliflozin beyond the trial duration such that dapagliflozin would become ineffective 5 years after trial completion. The base case assumed that dapagliflozin would reduce the risk of incident diabetes among individuals without diabetes at baseline by 32%^26^; a sensitivity analysis assumed no reduction in the risk of incident diabetes.

#### Safety, Pill-Related Disutility, and Treatment Discontinuation

As there were no observed differences in safety outcomes in the DAPA-HF trial,^4,11^ we did not model any additional costs or quality-of-life penalties caused by adverse drug events. As patients in the GDMT arm would already be receiving pills on a daily basis (either several times or twice daily), we did not model any additional pill-related disutility (ie, decrement in quality-of-life from taking a daily pill of dapagliflozin).^27^ The base case assumed that patients on dapagliflozin would be adherent to the therapy; a sensitivity analysis examined the effect of nonadherence by incorporating a 0.27% monthly probability of discontinuing dapagliflozin for the first 2 years (in order to replicate the 4.7% discontinuation observed in the DAPA-HF trial).^4^

#### Survival

The model incorporated a nonparametric survival model as follows. Over the first 18 months, the control arms replicated the survival observed in the DAPA HF trial (separately for patients with and without diabetes at baseline). Next, we compared the observed mortality rate in the control arms in the last 6 months of the trial with the mortality rate in the age-matched US general population, yielding a mortality rate ratio of 8.66 in patients with HFrEF and diabetes and 4.88 in patients with HFrEF but without diabetes at baseline.^28^ This rate ratio was applied to the age-specific mortality in the general US population to estimate the age-specific survival in the control arms beyond trial completion (eFigure 1 in the Supplement). A sensitivity analysis assumed more favorable long-term survival in the control arm based on a recently published analysis that pooled the control arms of several contemporary HFrEF trials (eFigure 1 in the Supplement).^29^

The age-specific mortality rate in the intervention arms was estimated by applying the hazard ratio for all-cause mortality observed in the DAPA-HF trial to the control survival curves (separately for patients with or without diabetes). Additional modeling details are available in eMethods in the Supplement.

### Costs

Manufacturer discounts and rebates for diabetes therapies such as SGLT2 inhibitors have been particularly large in recent years,^7^ so that the net price paid by payers is substantially lower than the list price. As recommended by the Second Panel on Cost-Effectiveness, our base case incorporated the drug cost reported in the Federal Supply Schedule ($4192 for a year’s supply in August 2020).^10,15^ In deterministic sensitivity analyses, we varied the annual cost of dapagliflozin from $953 (a heavily discounted net price at which dapagliflozin is available to some US payers) to $6188 (the wholesale acquisition cost).^16^ We also identified the cost at which the addition of dapagliflozin to GDMT would become cost-effective at thresholds of $50 000, $100 000, and $150 000 per quality-adjusted life-year (QALY) gained (additional details regarding the estimation of costs are provided in the eMethods and eTable 1 in the Supplement). Patients with diabetes were assumed to have higher background health care costs than patients without diabetes. All costs were inflated to 2020 US dollars using the medical component of the Personal Consumption Expenditure Index.^10,30,31^

### Quality-of-Life Estimates

The Kansas City Cardiomyopathy Questionnaire overall summary score (KCCQ-OSS) was used in the DAPA-HF trial to measure the heart failure–specific health status of patients at baseline and at 4-month and 8-month follow-up.^4,11,21,32^ To translate KCCQ-OSS to quality-of-life weights, we used an algorithm developed by Spertus et al^19^ that maps individual-level KCCQ scores to EQ-5D–based health-related quality-of-life estimates (eTables 2 and 3 in the Supplement). The base case replicated quality-of-life estimates reported in the DAPA-HF trial and then modeled an age-adjusted decline beyond the end of the trial period based on the community-based preference scores derived from the Medical Expenditure Panel Survey.^22^ The model applied short-term quality-of-life tolls for heart failure hospitalizations and urgent heart failure visits, and a sustained penalty for incident diabetes.^22,23,33^

### Statistical Analysis

Our primary outcome was the incremental cost-effectiveness ratio (ICER) of adding dapagliflozin to GDMT compared with GDMT alone (in US dollars per life-year gained and US dollars per QALY gained), for the entire cohort and stratified by baseline diabetes status. We ran 10 000 simulations using input parameter values randomly drawn from prespecified statistical distributions to generate 95% uncertainty intervals (UI) for key outcome measures and to determine the proportion of simulations in which dapagliflozin would be cost-effective at varying willingness-to-pay thresholds. We assumed a cost-effectiveness threshold of $100 000 per QALY gained,^34^ but also examined the value of the intervention per the cost/value methodology recommendations of the American College of Cardiology and American Heart Association (high-value, less than $50 000 per QALY gained; intermediate-value, $50 000 or more to less than $150 000 per QALY gained; and low-value, $150 000 or more per QALY gained).^35^ In addition to the probabilistic analyses described above, we performed deterministic sensitivity analyses to reflect uncertainty in key input parameters by varying 1 input at a time across the range specified in Table 1 while holding all other parameters at their base-case values.

## Results

The simulated cohort in the model had a starting age of 66 years, and 41.8% of patients in the simulation had diabetes at baseline. Median (interquartile range) undiscounted survival in the GDMT arm was 6.8 (3.5-11.3) years (patients without diabetes, 7.6 [3.9-12.3] years; patients with diabetes, 5.7 [3.0-9.9] years). The model replicated the rates of all-cause mortality, HFrEF hospitalization, and urgent heart failure visits observed in the DAPA-HF trial (eTable 4 in the Supplement).

### Base Case

**Figure 2.**
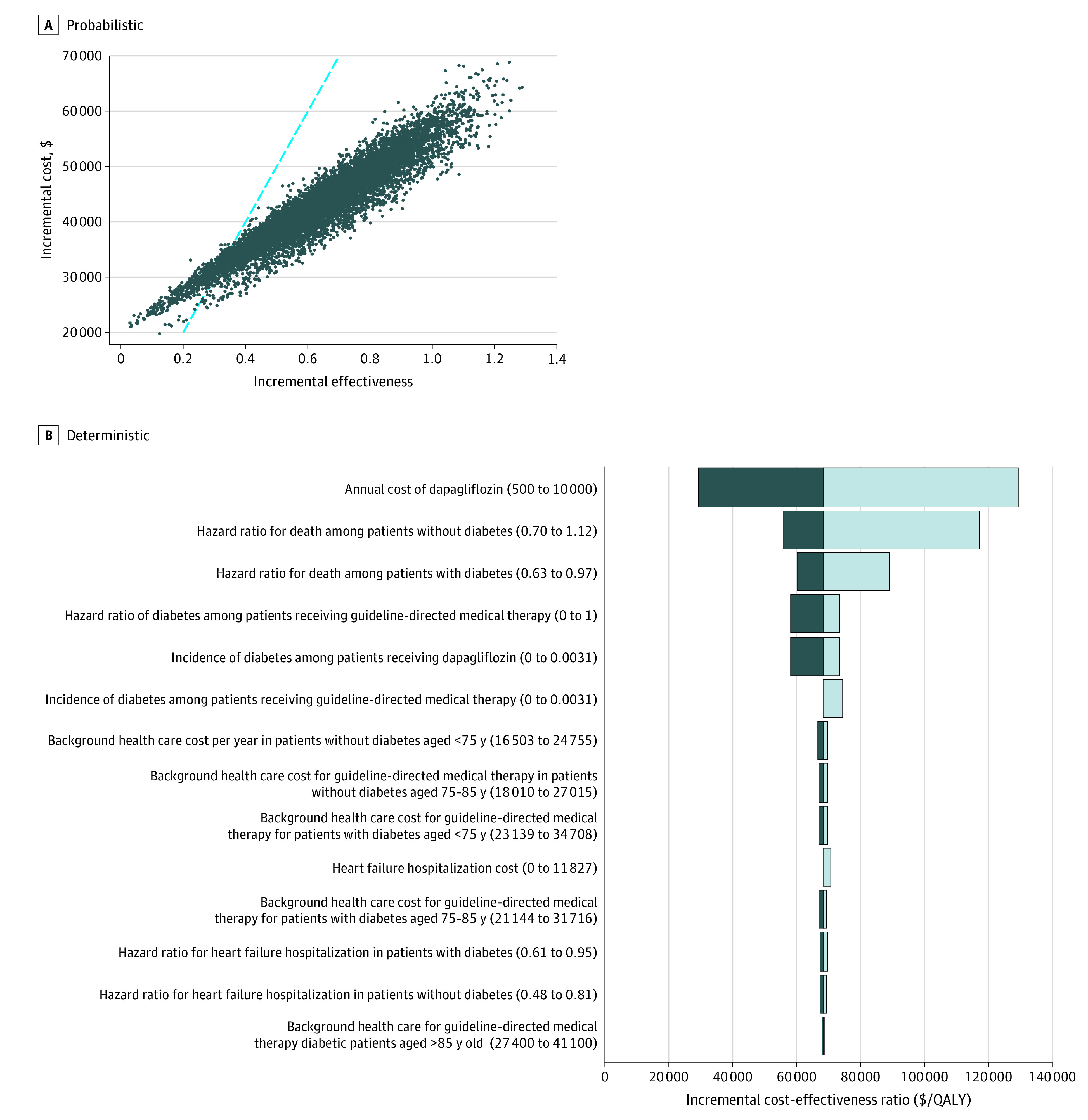
Sensitivity Analyses In panel A, the blue dashed line indicates the assumed cost-effectiveness threshold of $100 000 per quality-adjusted life years (QALYs) gained (blue dashed line). Adding dapagliflozin to GDMT was cost-effective in 94% of 10 000 probabilistic simulations.

Adding dapagliflozin to GDMT in patients with HFrEF was projected to lower the rate of HFrEF hospitalizations from 0.10 (95% CI, 0.09-0.11) to 0.07 (95% CI, 0.06-0.08) per person-year and improve quality-adjusted survival by 0.63 (95% uncertainty interval [UI], 0.25-0.94) QALYs (Table 2). Patients receiving dapagliflozin incurred $27 700 (95% UI, $25 700-$29 800) in lifetime spending on dapagliflozin, which was only partially offset by savings resulting from reduced HFrEF hospitalizations. After accounting for increased health care costs related to prolonged survival, the intervention arm had a net increase in lifetime health care costs of $42 800 (95% UI, $37 100-$50 300). As a result, adding dapagliflozin to GDMT had an ICER of $68 300 per QALY gained (95% UI, $54 600-$117 600 per QALY gained) compared with GDMT alone, and was cost-effective in 94% of 10 000 probabilistic simulations (Table 2 and Figure 2).^36^ Subgroup analyses demonstrated similar ICERs among patients without or with diabetes (without diabetes: $69 600 [95% UI, $50 700-$445 700] per QALY gained; cost-effective in 84% of 10 000 probabilistic simulations; with diabetes: $66 800 [95% UI, $53 800-$116 600] per QALY gained; cost-effective in 95% of 10 000 probabilistic simulations) (Table 2).

**Table 2.** Base Case Results

Characteristic	All patients		No diabetes		Diabetes	
	GDMT	Dapagliflozin	GDMT	Dapagliflozin	GDMT	Dapagliflozin
Health outcomes						
Survival, life-years (undiscounted)	6.82 (6.77-6.86)	7.73 (7.10-7.76)	7.60 (7.51-7.68)	8.42 (7.57-9.28)	5.73 (5.38-6.13)	6.77 (5.59-8.18)
Survival, life-years (discounted)	5.91 (5.87-5.91)	6.6 (6.13-7.10)	6.52 (6.46-6.57)	7.12 (6.48-7.77)	5.07 (4.79-5.39)	5.88 (4.96-6.96)
Incremental life years (discounted)	[Reference]	0.64 (0.21-1.11)	[Reference]	0.61 (0.18-1.13)	[Reference]	0.81 (0.22-1.45)
QALYs (discounted)	4.73 (4.69-4.76)	5.36 (4.98-5.76)	5.28 (5.23-5.33)	5.86 (5.33-6.38)	3.96 (3.77-4.24)	4.66 (3.96-5.56)
Incremental QALYs (discounted)	[Reference]	0.63 (0.25-0.94)	[Reference]	0.58 (0.21-0.98)	[Reference]	0.70 (0.23-1.20)
evLYG^a^	[Reference]	0.76 (0.29-1.26)	[Reference]	0.69 (0.04-1.31)	[Reference]	0.87 (0.28-1.51)
Direct health care costs, $^b^						
Lifetime health care costs (discounted)	150 600 (131 300-172 200)	193 400 (168 400-222 500)	148 900 (126 000-174 700)	189 000 (158 800-224 400)	152 900 (126 000-174 700)	199 400 (163 400-224 400)
Spending on dapagliflozin	NA	27 700 (25 700-29 800)	NA	29 900 (27 200-32 600)	NA	24 700 (22 200-27 300)
Spending on HF hospitalizations	6900 (5200-9200)	5400 (3900-7500)	6600 (4900-8800)	4600 (3200-6600)	7300 (5500-9800)	6500 (4500-9300)
Incremental health costs (discounted)	[Reference]	42 800 (37 100-50 300)	[Reference]	40 100 (32 700-49 700)	[Reference]	46 500 (32 700-49 700)
ICER, $						
Per life-year gained	[Reference]	61 800 (47 500 131 700)	[Reference]	66 200 (45 000-dominated)	[Reference]	57 300 (44 800-123 800)
Per QALY gained	[Reference]	68 300 (54 600-117 600)	[Reference]	69 600 (50 700-445 700)	[Reference]	66 800 (53 800-116 600)
Per evLYG^a^	[Reference]	56 100 (44 700-100 700)	[Reference]	58 500 (42 300-315 600)	[Reference]	53 400 (42 800-97 200)

Abbreviations: evLYG, equal value of life-years gained; GDMT, guideline-directed medical therapy; HF, heart failure; ICER, incremental cost-effectiveness ratio; NA, not applicable; QALY, quality-adjusted life-year; UI, uncertainty interval.

^a^ As the use of QALYs may undervalue prolonged survival among individuals with imperfect quality-of-life at baseline, we also computed the incremental cost evLYG, an approach that assumes that any extension of life has a perfect quality-of-life.^36^

^b^ In 2020 US dollars.

### Sensitivity Analyses

**Figure 3.**
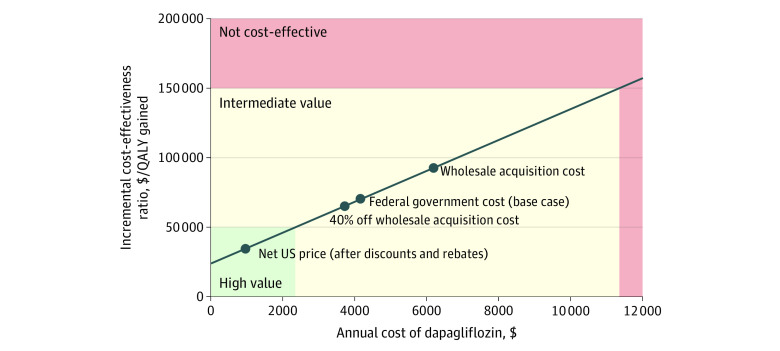
Incremental Cost-effectiveness of Dapagliflozin in Treatment of Heart Failure With Reduced Ejection Fraction We varied the annual cost of dapagliflozin, holding all other input parameters at their base-case value. The different annual costs analyzed included: (1) Federal Supply Schedule (base case, $4192); (2) wholesale acquisition price ($6188); (3) the price obtained if the wholesale acquisition were discounted by 40% (average value of rebates and discounts on diabetes pharmaceuticals) ($3713); and (4) a heavily discounted net price at which dapagliflozin is available in some US markets ($953). The color quadrants indicate the annual cost at which adding dapagliflozin to guideline-directed medical therapy would be cost-effective relative to guideline-directed medical therapy alone at thresholds of $50 000, $100 000, and $150 000 per quality-adjusted life year (QALY) gained. These results permit health systems and clinicians to estimate the incremental cost-effectiveness of dapagliflozin for HFrEF in the context of their cost of a year’s supply of dapagliflozin.

The cost-effectiveness of adding dapagliflozin to GDMT compared with GDMT alone was sensitive to the annual cost of dapagliflozin (Figure 3), the association of dapagliflozin with the risk of developing diabetes among individuals without diabetes at baseline, and the durability of effectiveness (Figure 2). For instance, if the annual cost of dapagliflozin were as low as $500, the ICER for dapagliflozin compared with GDMT alone would decline to $29 400 per QALY gained. If dapagliflozin had no effect on the rate of incident diabetes among individuals with diabetes at baseline, the ICER would increase to $73 500 per QALY gained. If the association of dapagliflozin with all-cause mortality were to reduce linearly for 5 years after trial completion, the ICER would increase to $89 300 per QALY gained. The use of dapagliflozin would be cost-effective at $50 000, $100 000, and $150 000 per QALY gained at annual costs of $2500 (40.4% lower than base-case price and 59.6% lower than list price), $7200 (72% higher than base-case price and 16% higher than list price), and $11 900 (183% higher than base-case price and 92% higher than list price), respectively (Figure 3). Additional results from sensitivity analyses are presented in the eMethods, eTables 5 and 6, and eFigure 2 in the Supplement.

## Discussion

In a simulation model of HFrEF in the US, we found that the addition of dapagliflozin to GDMT was projected to improve quality-adjusted survival in patients with HFrEF, and its use would be cost-effective relative to GDMT alone at 2020 Federal Supply Schedule prices. The lifetime ICER of $68 300 per QALY gained compared with GDMT alone would make the use of dapagliflozin for the treatment of HFrEF an “intermediate-value” intervention per the American College of Cardiology/American Heart Association Cost/Value framework.^35^ Remarkably, the ICER was similar in patients with or without diabetes at baseline. This is because, compared with patients without diabetes, patients with diabetes receive a larger clinical benefit (given their higher baseline risk of major adverse cardiovascular events) but also accrue higher health care costs for every year of prolonged survival. The observation that dapagliflozin therapy was cost-effective in 94% of 10 000 probabilistic simulations suggests that our findings are robust across a wide range of estimates of key model parameters.

To our knowledge, this is the first systematic cost-effectiveness analysis of dapagliflozin for patients with HFrEF from a US health care sector perspective. Prior analyses performed from the perspective of other health systems (including UK, Germany, Spain, China, and Australia) also projected dapagliflozin to be cost-effective in those health systems, but substantial cross-country differences in drug and health care costs and HFrEF outcomes preclude direct comparisons.^37,38,39^ For instance, our estimates of long-term effectiveness of dapagliflozin (a gain of 0.63 QALYs) is comparable to the gain of 0.48 to 0.50 QALYs in the British and European models, but somewhat higher than the gains in the Chinese (0.38) and Australian (0.29) models. The annual cost of dapagliflozin is substantially higher in the US compared with other health systems ($4192 in our base case, compared with the equivalent of $302-$780 in Europe, $257 in China, and $563 in Australia per the published analyses).^37,38,39^ Furthermore, we included all background health care costs (in line with current recommendations) whereas the European analysis only included costs related to diabetes and heart failure.^10^ As a result, despite comparable health gains, the ICER was substantially higher in our analysis ($54 600-$117 600 per QALY gained compared with $6343-$11 091 per QALY gained in Europe, $3828 per QALY gained in China, and $9550 per QALY gained in Australia).

Cost-effectiveness analysis provides a formal mechanism to quantify the gains or losses in population health as a result of a particular intervention.^40^ Treatments for HFrEF may potentially have an outsized impact on health care budgets because a large number of patients may be eligible for lifelong therapy, which necessitates the prioritization of cost-effective therapies. However, the slow initial uptake of sacubitril-valsartan suggests that evidence of cost-effectiveness alone cannot surmount the constellation of patient-, physician-, and health system–related barriers that can impede widespread adoption of new therapies.^5,41,42^ Key among them is the fragmented US health care system, where financial incentives for various stakeholders are frequently misaligned. For instance, pharmaceutical and medical coverage may be offered by different payers, so that the pharmaceutical plan does not have the incentive to pay for high-cost medications that prevent hospitalizations, the savings of which accrue to the medical plan. Even when insurers are responsible for both medical and pharmaceutical benefits, as with many commercial insurers and Medicare Advantage plans, payers may not benefit from savings that occur several years in the future, at which point the patient may have switched to a different plan. As a result, payers frequently impose prior authorization requirements or high copayments to restrict uptake of high-cost therapies. Copayments are typically based on the list price of the drug before any manufacturer discounts and vary from month-to-month based on the benefit structure of the insurance plan^7,43^; they therefore pose a substantial financial burden on patients and adversely affect persistence. Until policy reforms address the misalignment of financial incentives for payers in the US health system and cap or eliminate copayments for cost-effective therapies (as in recent proposals to expand access to insulin),^44^ clinicians should discuss medication affordability with their patients at regular intervals in order to preempt cost-related nonadherence.

Although total and out-of-pocket costs have slowed uptake of novel cardiovascular therapies in the recent past,^19,43,45,46^ 3 factors justify our cautious optimism that the experience with SGLT2 inhibitors will be different. First, diabetes therapies, including SGLT2 inhibitors, receive some of the largest manufacturer discounts observed in the US pharmaceutical market, and these discounts have fully offset increases in list prices in recent years.^7^ Second, the availability of several SGLT2 inhibitors with proven benefit in HFrEF treatment may allow payers to negotiate better prices in return for preferential formulary placement.^5,47^ As a result, the majority of US payers will be able to continue to access at least 1 SGLT2 inhibitor at a reasonable price. Finally, recently proposed policy reforms would force payers to pass on at least some of the manufacturer discounts to patients.^7^ Collectively, these changes could substantially lower total costs for payers and out-of-pocket costs for patients, and expand access to this cost-effective therapy.

### Limitations

This study had several limitations. The efficacy and safety of dapagliflozin were estimated from a single randomized clinical trial with a mean follow-up of 18 months. We estimated long-term survival based on a combination of trial and vital statistics data, and examined alternative survival models in sensitivity analyses, but our results should be updated when data from longer follow-up become available. We did not examine heterogeneity other than by diabetes status (eg, by New York Heart Association symptom class). However, the goal of this study was to evaluate population-level effects on health and health care spending, which are adequately reflected in our use of population-level estimates of risk, benefit, and cost. If future studies demonstrate that treatment with dapagliflozin reduces the need for kidney replacement therapy in patients with HFrEF, our model would have underestimated the economic benefits of SGLT2 inhibitor therapy.^48^ The price of dapagliflozin is likely to decline after expiration of the market exclusivity period and entry of multiple generics. We modeled contemporary US prices so that our findings could inform current pricing and uptake. We explored a wide range of drug prices in sensitivity analyses, but our findings should be updated when new data on pricing, effectiveness, or safety become available. We used a novel approach to convert KCCQ-OSS to health-related quality-of-life. However, any bias in the conversion would not have materially affected our findings as it would affect both the control and intervention arms, and the health gains in the dapagliflozin arm were primarily because of improved survival (rather than from improved quality of life).

## Conclusions

In a simulation model calibrated to the results of the DAPA-HF trial, adding dapagliflozin to GDMT was projected to produce substantial clinical gains in patients with HFrEF, with an acceptable increase in associated costs. At a willingness-to-pay threshold of $100 000 per QALY gained, treatment with dapagliflozin, at an annual cost of $4192, would be cost-effective therapy in patients with HFrEF regardless of whether treated patients have diabetes. Scalable strategies to ensure affordable access to and widespread uptake of dapagliflozin are urgently needed.

## Notes

Supplement. eMethods. **eTable 1.** Detailed Cost of Items Included in Calculation of Urgent Heart Failure Visit Cost **eTable 2.** Mapping KCCQ-OSS to Health-related Quality-of-Life Weights **eTable 3.** Improvement in Quality-of-Life with Dapagliflozin Therapy Stratified by Baseline Diabetes Status—In Trial Observations **eTable 4.** Model Calibration **eTable 5.** Sensitivity Analyses of Alternative Survival and Effect of Dapagliflozin on All-Cause Mortality **eTable 6.** Sensitivity Analyses of Alternative Survival and Effect of Dapagliflozin on All-Cause Mortality **eFigure 1.** Survival Model, Extrapolation Beyond Trial Duration Used in the Base Case and Alternative Survival Model for Sensitivity Analysis **eFigure 2.** Acceptability Curves eReferences.

## References

[1] Wiviott SD, Raz I, Bonaca MP, ; DECLARE–TIMI 58 Investigators. Dapagliflozin and cardiovascular outcomes in type 2 diabetes. N Engl J Med. 2019;380(4):347-357. doi:10.1056/NEJMoa1812389 30415602

[2] Neal B, Perkovic V, Mahaffey KW, ; CANVAS Program Collaborative Group. Canagliflozin and cardiovascular and renal events in type 2 diabetes. N Engl J Med. 2017;377(7):644-657. doi:10.1056/NEJMoa1611925 28605608

[3] Zinman B, Wanner C, Lachin JM, ; EMPA-REG OUTCOME Investigators. Empagliflozin, cardiovascular outcomes, and mortality in type 2 diabetes. N Engl J Med. 2015;373(22):2117-2128. doi:10.1056/NEJMoa1504720 26378978

[4] McMurray JJV, Solomon SD, Inzucchi SE, ; DAPA-HF Trial Committees and Investigators. Dapagliflozin in patients with heart failure and reduced ejection fraction. N Engl J Med. 2019;381(21):1995-2008. doi:10.1056/NEJMoa1911303 31535829

[5] Packer M, Anker SD, Butler J, ; EMPEROR-Reduced Trial Investigators. Cardiovascular and renal outcomes with empagliflozin in heart failure. N Engl J Med. 2020;383(15):1413-1424. doi:10.1056/NEJMoa2022190 32865377

[6] US Food and Drug Administration. FDA approves new treatment for a type of heart failure. US Food and Drug Administration press release. Published May 5, 2020. Accessed June 21, 2021. https://www.fda.gov/news-events/press-announcements/fda-approves-new-treatment-type-heart-failure

[7] Hernandez I, San-Juan-Rodriguez A, Good CB, Gellad WF. Changes in list prices, net prices, and discounts for branded drugs in the US, 2007-2018. JAMA. 2020;323(9):854-862. doi:10.1001/jama.2020.1012 32125403 PMC7054846

[8] Yancy CW, Jessup M, Bozkurt B, . 2017 ACC/AHA/HFSA focused update of the 2013 ACCF/AHA guideline for the management of heart failure: a report of the American College of Cardiology/American Heart Association Task Force on Clinical Practice Guidelines and the Heart Failure Society of America. Circulation. 2017;136(6):e137-e161. doi:10.1161/CIR.0000000000000509 28455343

[9] Ponikowski P, Voors AA, Anker SD, ; ESC Scientific Document Group. 2016 ESC guidelines for the diagnosis and treatment of acute and chronic heart failure: the Task Force for the Diagnosis and Treatment of Acute and Chronic Heart Failure of the European Society of Cardiology (ESC), developed with the special contribution of the Heart Failure Association (HFA) of the ESC. Eur Heart J. 2016;37(27):2129-2200. doi:10.1093/eurheartj/ehw128 27206819

[10] Sanders GD, Neumann PJ, Basu A, . Recommendations for conduct, methodological practices, and reporting of cost-effectiveness analyses: second panel on cost-effectiveness in health and medicine. JAMA. 2016;316(10):1093-1103. doi:10.1001/jama.2016.12195 27623463

[11] Petrie MC, Verma S, Docherty KF, . Effect of dapagliflozin on worsening heart failure and cardiovascular death in patients with heart failure with and without diabetes. JAMA. 2020;323(14):1353-1368. doi:10.1001/jama.2020.1906 32219386 PMC7157181

[12] Wadhera RK, Joynt Maddox KE, Wasfy JH, Haneuse S, Shen C, Yeh RW. Association of the hospital readmissions reduction program with mortality among Medicare beneficiaries hospitalized for heart failure, acute myocardial infarction, and pneumonia. JAMA. 2018;320(24):2542-2552. doi:10.1001/jama.2018.19232 30575880 PMC6583517

[13] Krumholz HM. Post-hospital syndrome—an acquired, transient condition of generalized risk. N Engl J Med. 2013;368(2):100-102. doi:10.1056/NEJMp1212324 23301730 PMC3688067

[14] Inzucchi SE, Docherty KF, Kober L, . Dapagliflozin and the incidence of type 2 diabetes in patients with heart failure and reduced ejection fraction: an exploratory analysis from DAPA-HF. Diabetes Care. 2021;44(2):586-594. 33355302 10.2337/dc20-1675

[15] US Department of Veterans Affairs. VA Federal Supply Schedule Service. Revised June 16, 2021. Accessed September 2020. https://www.va.gov/opal/nac/fss/pharmPrices.asp

[16] US Brand Rx WAC / Net Price. SSR Health website. Last updated August 6, 2019. Accessed August 2020. http://www.ssrhealth.com/research-archive/

[17] Agency for Healthcare Research and Quality. Healthcare Costs and Utilization Project—statistics on hospital stays. Accessed May 2020. https://hcupnet.ahrq.gov/

[18] Peterson C, Xu L, Florence C, Grosse SD, Annest JL. Professional fee ratios for US hospital discharge data. Med Care. 2015;53(10):840-849. doi:10.1097/MLR.0000000000000410 26340662 PMC4681390

[19] Kazi DS, Bellows BK, Baron SJ, . Cost-effectiveness of tafamidis therapy for transthyretin amyloid cardiomyopathy. Circulation. 2020;141(15):1214-1224. doi:10.1161/CIRCULATIONAHA.119.045093 32078382 PMC7156331

[20] US Centers for Medicare and Medicaid Services. Medicare Provider Utilization and Payment Data. Published 2017. Accessed May 2020. https://www.cms.gov/Research-Statistics-Data-and-Systems/Statistics-Trends-and-Reports/Medicare-Provider-Charge-Data/Physician-and-Other-Supplier

[21] Kosiborod MN, Jhund PS, Docherty KF, . Effects of dapagliflozin on symptoms, function, and quality of life in patients with heart failure and reduced ejection fraction: results from the DAPA-HF trial. Circulation. 2020;141(2):90-99. doi:10.1161/CIRCULATIONAHA.119.044138 31736335 PMC6964869

[22] Sullivan PW, Ghushchyan V. Preference-based EQ-5D index scores for chronic conditions in the United States. Med Decis Making. 2006;26(4):410-420. doi:10.1177/0272989X06290495 16855129 PMC2634296

[23] Jaagosild P, Dawson NV, Thomas C, ; SUPPORT Investigators. The Study to Understand Prognosis and Preferences for Outcomes and Risks of Treatments. Outcomes of acute exacerbation of severe congestive heart failure: quality of life, resource use, and survival. Arch Intern Med. 1998;158(10):1081-1089. doi:10.1001/archinte.158.10.1081 9605779

[24] AstraZeneca Pharmaceuticals LP. Farxiga (dapagliflozin) [package insert]. US Food and Drug Administration website. Revised May 2020. Accessed May 21, 2020. https://www.azpicentral.com/farxiga/farxiga.pdf

[25] US Food and Drug Administration. Farxiga (dapagliflozin) [package insert]. U.S. Food and Drug Administration website. Revised May 2020. Accessed May 21, 2020. https://www.accessdata.fda.gov/drugsatfda_docs/label/2020/202293s020lbl.pdf

[26] Inzucchi SE, Docherty K, Kober L, . Effect of dapagliflozin on the incidence of diabetes: a prespecified exploratory analysis from DAPA-HF. Diabetes. 2020;69(suppl 1):271-OR. doi:10.2337/db20-271-OR32079703

[27] Hutchins R, Viera AJ, Sheridan SL, Pignone MP. Quantifying the utility of taking pills for cardiovascular prevention. Circ Cardiovasc Qual Outcomes. 2015;8(2):155-163. doi:10.1161/CIRCOUTCOMES.114.001240 25648463

[28] National Center for Health Statistics. United States Life Tables, 2017. Published June 24, 2019. Accessed 2020. https://www.cdc.gov/nchs/products/life_tables.htm 32501200

[29] Vaduganathan M, Claggett BL, Jhund PS, . Estimating lifetime benefits of comprehensive disease-modifying pharmacological therapies in patients with heart failure with reduced ejection fraction: a comparative analysis of three randomised controlled trials. Lancet. 2020;396(10244):121-128. doi:10.1016/S0140-6736(20)30748-0 32446323

[30] US Bureau of Economic Analysis. Table 2.4.4. price indexes for personal consumption expenditures by type of product. Last revised July 31, 2021. Accessed May 2020. https://apps.bea.gov/iTable/iTable.cfm?reqid=19&step=3&isuri=1&nipa_table_list=69&categories=survey

[31] US Bureau of Economic Analysis. Table 2.8.7. percent change from preceding period in prices for personal consumption expenditures by major type of product, monthly. Last revised May 28, 2021. Accessed May 2020. https://apps.bea.gov/iTable/iTable.cfm?reqid=19&step=3&isuri=1&1921=survey&1903=84.

[32] Green CP, Porter CB, Bresnahan DR, Spertus JA. Development and evaluation of the Kansas City Cardiomyopathy Questionnaire: a new health status measure for heart failure. J Am Coll Cardiol. 2000;35(5):1245-1255. doi:10.1016/S0735-1097(00)00531-3 10758967

[33] Sandhu AT, Ollendorf DA, Chapman RH, Pearson SD, Heidenreich PA. Cost-effectiveness of sacubitril-valsartan in patients with heart failure with reduced ejection fraction. Ann Intern Med. 2016;165(10):681-689. doi:10.7326/M16-0057 27571284

[34] Vanness DJ, Lomas J, Ahn H. A health opportunity cost threshold for cost-effectiveness analysis in the United States. Ann Intern Med. 2021;174(1):25-32. doi:10.7326/M20-1392 33136426

[35] Anderson JL, Heidenreich PA, Barnett PG, . ACC/AHA statement on cost/value methodology in clinical practice guidelines and performance measures: a report of the American College of Cardiology/American Heart Association Task Force on Performance Measures and Task Force on Practice Guidelines. J Am Coll Cardiol. 2014;63(21):2304-2322. doi:10.1016/j.jacc.2014.03.016 24681044

[36] Nord E, Pinto JL, Richardson J, Menzel P, Ubel P. Incorporating societal concerns for fairness in numerical valuations of health programmes. Health Econ. 1999;8(1):25-39. doi:10.1002/(SICI)1099-1050(199902)8:1<25::AID-HEC398>3.0.CO;2-H 10082141

[37] McEwan P, Darlington O, McMurray JJV, . Cost-effectiveness of dapagliflozin as a treatment for heart failure with reduced ejection fraction: a multinational health-economic analysis of DAPA-HF. Eur J Heart Fail. 2020;22(11):2147-2156. doi:10.1002/ejhf.1978 32749733 PMC7756637

[38] Yao Y, Zhang R, An T, Zhao X, Zhang J. Cost-effectiveness of adding dapagliflozin to standard treatment for heart failure with reduced ejection fraction patients in China. ESC Heart Fail. 2020. doi:10.1002/ehf2.12844 33107212 PMC7754897

[39] Savira F, Wang BH, Kompa AR, . Cost-effectiveness of dapagliflozin in chronic heart failure: an analysis from the Australian healthcare perspective. Eur J Prev Cardiol. 2020;2047487320938272.doi:10.1177/2047487320938272 34402872

[40] Husereau D, Drummond M, Petrou S, ; CHEERS Task Force. Consolidated Health Economic Evaluation Reporting Standards (CHEERS) statement. Value Health. 2013;16(2):e1-e5. doi:10.1016/j.jval.2013.02.010 23538200

[41] McMurray JJ, Packer M, Desai AS, ; PARADIGM-HF Investigators and Committees. Angiotensin-neprilysin inhibition versus enalapril in heart failure. N Engl J Med. 2014;371(11):993-1004. doi:10.1056/NEJMoa1409077 25176015

[42] Greene SJ, Butler J, Albert NM, . Medical therapy for heart failure with reduced ejection fraction: the CHAMP-HF registry. J Am Coll Cardiol. 2018;72(4):351-366. doi:10.1016/j.jacc.2018.04.070 30025570

[43] DeJong C, Kazi DS, Dudley RA, Chen R, Tseng CW. Assessment of national coverage and out-of-pocket costs for sacubitril/valsartan under Medicare part D. JAMA Cardiol. 2019;4(8):828-830. doi:10.1001/jamacardio.2019.2223 31290933 PMC6624798

[44] Beran D, Hirsch IB, Yudkin JS. Why are we failing to address the issue of access to insulin? a national and global perspective. Diabetes Care. 2018;41(6):1125-1131. doi:10.2337/dc17-2123 29784696

[45] Kazi DS, Lu CY, Lin GA, . Nationwide coverage and cost-sharing for PCSK9 inhibitors among Medicare part D plans. JAMA Cardiol. 2017;2(10):1164-1166. doi:10.1001/jamacardio.2017.3051 28903137 PMC5815006

[46] Hlatky MA, Kazi DS. PCSK9 inhibitors: economics and policy. J Am Coll Cardiol. 2017;70(21):2677-2687. doi:10.1016/j.jacc.2017.10.001 29169476

[47] Bhatt DL, Szarek M, Steg PG, ; SOLOIST-WHF Trial Investigators. Sotagliflozin in patients with diabetes and recent worsening heart failure. N Engl J Med. 2021;384(2):117-128. doi:10.1056/NEJMoa2030183 33200892

[48] Heerspink HJL, Stefánsson BV, Correa-Rotter R, ; DAPA-CKD Trial Committees and Investigators. Dapagliflozin in patients with chronic kidney disease. N Engl J Med. 2020;383(15):1436-1446. doi:10.1056/NEJMoa2024816 32970396

