# Supplementary material for: Cost-effectiveness of Dapagliflozin for the Treatment of Heart Failure With Reduced Ejection Fraction
Source: JAMA Netw Open. 2021 Jul 27;4(7):e2114501. doi: 10.1001/jamanetworkopen.2021.14501 (PMC8317009; doi:10.1001/jamanetworkopen.2021.14501)
Supplement: Supplement. — eMethods. eTable 1. Detailed Cost of Items Included in Calculation of Urgent Heart Failure Visit Cost eTable 2. Mapping KCCQ-OSS to Health-related Quality-of-Life Weights eTable 3. Improvement in Quality-of-Life with Dapagliflozin Therapy Stratified by Baseline Diabetes Status—In Trial Observations eTable 4. Model Calibration eTable 5. Sensitivity Analyses of Alternative Survival and Effect of Dapagliflozin on All-Cause Mortality eTable 6. Sensitivity Analyses of Alternative Survival and Effect of Dapagliflozin on All-Cause Mortality eFigure 1. Survival Model, Extrapolation Beyond Trial Duration Used in the Base Case and Alternative Survival Model for Sensitivity Analysis eFigure 2. Acceptability Curves eReferences. [file jamanetwopen-e2114501-s001.pdf]

Supplemental Online Content

Isaza N, Calvachi P, Raber I, et al. Cost-effectiveness of dapagliflozin for the treatment of heart failure with reduced ejection fraction. *JAMA Netw Open.* 2021;4(7):e2114501. doi:10.1001/jamanetworkopen.2021.14501

eMethods.

eTable 1. Detailed Cost of Items Included in Calculation of Urgent Heart Failure Visit Cost

eTable 2. Mapping KCCQ-OSS to Health-related Quality-of-Life Weights

eTable 3. Improvement in Quality-of-Life with Dapagliflozin Therapy Stratified by Baseline Diabetes Status—In-trial Observations

eTable 4. Model Calibration.

eTable 5. Sensitivity Analyses of Alternative Survival and Effect of Dapagliflozin on All-Cause Mortality

eTable 6. Sensitivity Analyses of Alternative Survival and Effect of Dapagliflozin on All-Cause Mortality

eFigure 1. Survival Model, Extrapolation Beyond Trial Duration Used in the Base Case and Alternative Survival Model for Sensitivity Analysis

eFigure 2. Acceptability Curves

eReferences

This supplemental material has been provided by the authors to give readers additional information about their work.

© 2021 Isaza N et al. *JAMA Network Open.*

eMethods

Table of Contents

I. Additional Modeling Details.

a. Model overview

b. Transitional probabilities

c. Survival model

d. Cost of Dapagliflozin

e. Cost of Heart Failure Hospitalization and Urgent Heart Failure Visits

f. Background Healthcare Costs

g. Estimating Quality of Life Parameters

h. Main Outcomes Measures

i. Sensitivity Analyses

II. Model Calibration

III. Additional Results

a. Sensitivity of the ICER to Changes in Selected Variables

b. Sensitivity Analysis of Null- or Harmful Effect of Dapagliflozin on All-Cause Mortality

c. Sensitivity Analysis Assuming No Impact of Dapagliflozin on the Risk of Incident Diabetes

d. Sensitivity Analysis of Declining Effectiveness of Dapagliflozin

e. Sensitivity Analysis of Alternative Survival

f. Additional Probabilistic Sensitivity Analysis Results

g. Sensitivity Analysis of Drug Discontinuation

© 2021 Isaza N et al. *JAMA Network Open.*

h. Acceptability curves

I. Additional Modeling Details

a. Model overview

We developed a state-transition Markov model to compare two treatment strategies among patients with HFrEF: 1) GDMT, comprised of an angiotensin-converting–enzyme inhibitor, an angiotensin-receptor blocker, or an angiotensin receptor-neprilysin inhibitor, in addition to a beta-blocker and a mineralocorticoid receptor antagonist;[1,2] and 2) dapagliflozin added to GDMT. Each treatment arm was further divided in two subgroups depending on the diabetes status at baseline. In monthly cycles, patients could continue to live with HFrEF, have an urgent care visit for a heart failure exacerbation, be hospitalized for a heart failure exacerbation, or die from cardiovascular or non-cardiovascular causes. Patients who survived the index HF hospitalization were at increased risk of repeat hospitalization in the month following the index event. The model included patients with or without diabetes; patients who did not have diabetes at baseline could develop diabetes during follow-up. Patients who survived a heart failure hospitalization were at increased risk of rehospitalization and death over the following 30 days.

With the exception of death and incident diabetes, the model allowed for recurrent events.

b. Transitional Probabilities

Key model inputs were derived from published primary and secondary analyses of the DAPA-HF, U.S. regulatory review documents that are publicly available through the U.S. FDA, published clinical and epidemiologic studies, national claims data, and the Medical

© 2021 Isaza N et al. *JAMA Network Open.*

Expenditure Panel Survey.[3-14] Key input parameters are summarized in Table 1 from the manuscript.

Key transitional probabilities include the rate of death from any cause (Rate of death from any cause, per person year [first 24 months]), rate of heart failure hospitalization (Rate of HF hospitalizations, per person year), heart failure urgent care visits (Rate of urgent HF visits, per person year) to which patients with and without diabetes would be subjected in monthly cycles. For patients with an admission there was a risk for death during that specific cycle (Proportion of HF hospitalizations that are fatal). Additionally, patients with admission for HF would transiently switch into a different state for the next cycle in which there was increased risk of readmission (Probability of 30-day readmission after a HF hospitalization and Proportion of HF-specific readmissions), Finally patient without diabetes at baseline had a monthly risk of incident diabetes (Rate of incident diabetes, per person year) and this event would trigger switchint into the the state of patient with diabetes at baseline.

In particular, the probability of heart failure readmissions was derived from published literature. In an analysis of Medicare data by Wadhera et.al, the 30-day heart failure readmission after a heart failure hospitalization was approximately 20%.[7] Krumholz and colleagues note that 37% of readmissions that occur within 30 days of discharge after a heart failure hospitalization are for a heart failure exacerbation.[15] Our model used these inputs to simulate the 30-day readmissions for heart failure.

c. Survival Model

The survival model was developed using the following non-parametric approach:

© 2021 Isaza N et al. *JAMA Network Open.*

i. In a subgroup analysis of the DAPA-HF trial, Petrie et al. reported the trial outcomes stratified by diabetes status at baseline.[4] We first digitized the Kaplan Meier curves for all-cause mortality using WebPlotDigitizer 4.3 (Pacifica, CA). These data were used to estimate the monthly rate of all-cause mortality in the control arm, separately for patients without and with diabetes at baseline. In eAppendix Figure 1, panel A, the solid orange line represents survival among patients without diabetes at baseline and the solid blue line represents the survival of patients with diabetes at baseline as observed in the DAPA-HF trial.

ii. We computed the mortality rate of patients in the control arm during the last 6 months of the trial (0.039 in patients without and 0.069 in patients with diabetes at baseline).

iii. We estimated the mortality rate for the age-matched US population using the US Life Tables published by the National Center for Health Statistics (for ages 67.5 to 68 years).[16] In eAppendix Figure 1, panel B, the solid black line represents the survival of the general US population starting at age 66. Given that the US Life tables are constructed based on population-wide mortality statistics, we address the limitation derived from assumptions of linear life expectancy.

iv. Next, we compared the mortality rates in the control arms of the subgroup analysis of the DAPA-HF trial to the mortality rate in the US general population. This yielded rate ratios of 8.66 for patients with diabetes and 4.88 for patients without diabetes. In other words, patients with diabetes in

the control arm of DAPA-HF had a mortality rate that was 8.66-fold that observed among 67.5-68-year-olds in the US population.

v. We assumed that this increased mortality would be sustained beyond the duration of the trial period. We applied the rate ratio computed in step iv above to the survival in the general US population (eAppendix Figure 1, Panel C). This was done separately for each subgroup based on diabetes status at baseline. In eAppendix Figure 1, panel D, the dashed orange line represents the beyond-trial survival extrapolated using this approach in patients without diabetes at baseline and the dashed blue line represents the beyond-trial survival extrapolated using this approach in patients with diabetes at baseline.

vi. In each case, all-cause mortality in the intervention arm was estimated by applying the rate ratio for all-cause mortality for dapagliflozin 0.78 for patients with diabetes, and 0.88 for patients without diabetes) to the corresponding age-matched mortality rate in the control arm.

vii. In sensitivity analyses, we modeled a more optimistic estimate of long-term survival based on a recently published analysis that pooled trial-level data from 3 contemporary trials of HFrEF.[3,17-19] In this study by Dr. Vaduganathan and colleagues, the investigators projected absolute survival gains with comprehensive disease-modifying pharmacological therapy if applied long term, compared with conventional therapies.[17] Patients in the control arm of the studies were assumed to receive at least and angiotensin-converting–enzyme inhibitor, or an angiotensin-receptor

blocker, or and a beta-blocker at the recommended or maximal tolerated dose. We used these data to model survival in the control arm in a scenario analysis. In eAppendix Figure 1, panel E, the solid red line shows the survival in patients with diabetes at baseline and solid green line shows the survival in patients without diabetes at baseline, using data from the study by Vaduganathan and colleagues.

d. Cost of Dapagliflozin

In the U.S. pharmaceutical market, the actual drug price faced by payors is obscured by confidential discounts and rebates offered by manufacturers. As a result, the Second Panel of Cost-Effectiveness in Health and Medicine recommended that the reference case assume a drug price equivalent to that in publicly available Federal Supply Schedule.[20] In the base case, we assumed an annual cost of dapagliflozin therapy of $4,192 per the August 2020 Federal Supply Schedule (FSS) “Big 4” pricing. This is the price paid by the four largest federal purchasers of drugs - the Department of Veterans Affairs, the Department of Defense, the Public Health Service, and the Coast Guard in August 2020).[21,22] We varied this input widely in sensitivity analyses from $953, a highly discounted net price available in some US markets according to SSR Health, a source of discount and net pricing data used in the drug pricing literature, to $6,188, the list price in August 2020.[23,24] We also computed the cost at which the addition of dapagliflozin to GDMT would become cost-effective at cost-effectiveness thresholds of $50,000, $100,000, and $150,000 per quality-adjusted life year (QALY).

e. Cost of Heart Failure Hospitalizations and Urgent Heart Failure Visits

The cost of heart failure hospitalizations was estimated from the 2017 Medicare Provider Utilization and Payment Data using the following Medicare Severity Diagnosis-Related Group (MS-DRG) codes[25]:

291 - HEART FAILURE & SHOCK W MCC

292 - HEART FAILURE & SHOCK W CC

293 - HEART FAILURE & SHOCK W/O CC/MCC

To address uncertainty in this parameter, in sensitivity analyses we defined the upper bound as the reimbursement for the relevant MS-DRG "with major complications" and the lower bound as the reimbursement for the relevant MS-DRG "without complications".

The cost of an urgent visit for heart failure is not well defined in the literature. We estimated the mean cost of an urgent heart failure visit at the Direct Access Cardiac Care Unit in our institution. We assumed that every urgent heart failure visit included a venipuncture, one set of laboratory studies (complete blood count, blood chemistry with electrolytes, kidney function tests, and brain natriuretic peptide), one electrocardiogram, and professional charges. Based on expert input, we assumed that 80% of visits require an intravenous infusion of furosemide after the initial bolus dose; the remainder have an adequate response to the intravenous bolus of furosemide alone. Based on expert input and our own institutional experience over the past one year, we assumed that 50% of patients undergo a chest x-ray, and 20% undergo transthoracic echocardiography during that visit. We collected the mean charges for services typically provided during an urgent care visit and applied a cost-center-specific charge-to-payment ratio of 39% from an

© 2021 Isaza N et al. *JAMA Network Open.*

ambulatory cost center to estimate the cost of the urgent care visit. eAppendix Table 1 shows detailed costs of charges included in the calculation of the urgent heart failure visit cost.

f. Background Healthcare Costs

We estimated background healthcare costs, defined as all direct medical costs, excluding heart failure hospitalization, urgent heart failure visits, and dapagliflozin costs, as the adjusted, survey-weighted mean total expenditures for individuals with history of heart failure from the 2006-2015 Medical Expenditure Panel Survey. They were stratified by age and diabetes status and excluding patients with cardiovascular hospitalizations in the survey year or the prior year.[26] This avoided double-counting of heart failure-related inpatient costs, which were modeled separately as noted above.

g. Quality-of-Life Parameters

The Kansas City Cardiomyopathy Questionnaire overall summary score (KCCQ-OSS) was used in DAPA-HF to measure heart failure-specific health status at baseline and at 4 and 8-month follow-up.[3,4,10,27] The KCCQ-OSS is a self-administered, 23-item questionnaire that quantifies physical limitations, symptoms, self-efficacy, social interference, and quality of life. The overall summary score ranges from 0 to 100 with higher scores indicating fewer symptoms, and a change of 5 or more points is considered clinically meaningful.[28] The mean (± standard deviation) baseline KCCQ-OSS was similar in both the GDMT and GDMT + dapagliflozin arms and was estimated based on the study by Kosiborod et. al to be 68.6 in the control arm and 68.4 in the intervention arm.[10] Over the course of 8-month follow up KCCQ-OSS improved in both arms to 72.7

© 2021 Isaza N et al. *JAMA Network Open.*

and 75.0 respectively with a statistically significant delta between the two arms.[10] To translate KCCQ-OSS to quality-of-life weights, we used an algorithm developed by John Spertus and colleagues that maps individual-level KCCQ scores to EQ-5D-based health-related quality-of-life estimates.[29] This algorithm, derived by comparing changes in objective heart failure measure with clinically observed changes over 6 ± 2 weeks in a cohort of 476 outpatients, has been validated in other datasets (John Spertus, personal communication, August 2020).[11,29]

As in our prior work, we used a linear regression model to identify the relationship between KCCQ-OS and EQ5D-derived utility weights, using the model

Health state utilities = a + b * KCCQ-OSS

We used previously estimated and validated mapping parameters a (intercept) and b (slope) to convert observed KCCQ-OSS values from DAPA-HF to quality-of-life weights for the model (eAppendix Table 2).[11] eAppendix Table 3 shows the quality-of-life weight changes in the base case analysis over the duration of the trial in each arm.

The value observed at the 8th month of follow-up was the starting value used to model an age-adjusted decline in in subsequent cycles. This was based on the community-based preference scores derived from the Medical Expenditure Panel Survey.[12] This included an additional adjustment for increase in the number of chronic conditions with age to account for the increasing comorbidity burden in an aging population.

© 2021 Isaza N et al. *JAMA Network Open.*

Additionally, we applied short-term quality-of-life tolls for heart failure hospitalizations and urgent heart failure visits as well as a sustained quality-of-life penalty for a diagnosis of diabetes of 0.0351 based on the community-based preference scores derived from the Medical Expenditure Panel Survey.[12]

h. Main Outcomes Measures

The primary outcome of our study was the incremental cost-effectiveness ratio (ICER) of adding dapagliflozin to GDMT compared with GDMT alone (in U.S. dollars per life-year gained and U.S. dollars per QALY gained). Because the use of QALYs may undervalue prolonged survival among individuals with imperfect quality-of-life at baseline, we also computed the incremental cost per equal value of life years gained (evLYG), an approach that assumes that any extension of life has a perfect quality-of-life.[30]

i. Sensitivity Analyses

As would be expected, secondary analyses of the DAPA-HF study cohort stratified by diabetes status yielded estimates that were less precise than those derived from the primary analysis that included the entire study cohort. Although formal tests of heterogeneity by diabetes did not suggest differences in effect size by diabetes status, when an outcome did not achieve statistical significance in stratified analyses but did so in the entire trial cohort (e.g., all-cause mortality in patients without diabetes at baseline and urgent heart failure visits in patients with diabetes at baseline), we performed additional deterministic sensitivity analyses that examined the effect of a null- or harmful effect of dapagliflozin on these outcomes.[4]

© 2021 Isaza N et al. *JAMA Network Open.*

II. Model Calibration

For model calibration, we performed 10,000 first-order microsimulations of the model to estimate event rates at 18 months and compared absolute rates of events (in the control and dapagliflozin arms) to published results from the DAPA-HF trial. We compared results for the entire study cohort as well as stratified by diabetes status at baseline.

For instance, all-cause mortality among patients receiving GDMT was 13.0% at 18 months in the model compared with 13.1% in the DAPA-HF trial.[3] The rate ratio for mortality among patients receiving dapagliflozin and GDMT compared with those receiving GDMT alone was 0.85 in the model cohort compared with a hazard ratio of 0.83 (95% CI 0.71 to 0.97) in the DAPA-HF trial.[3]

Similarly, the rate of heart failure hospitalizations among patients receiving GDMT in the model was 0.10 per patient year at 18 months compared with 0.10 per person year in the DAPA-HF trial.[3] The rate ratio for heart failure hospitalizations in patients receiving dapagliflozin and GDMT compared with those receiving GDMT alone was 0.70 in the model cohort compared with a hazard ratio of 0.70 (95% CI 0.59 to 0.83) in the DAPA-HF trial.[3]

Specifically, when stratified by baseline diabetes status, event rates were comparable in the model compared with the DAPA-HF trial, and rate ratios observed in the model (comparing outcomes in patients receiving dapagliflozin and GDMT with those receiving GDMT alone) were similar to the corresponding hazard ratios observed in the DAPA-HF trial (eAppendix Table 4).[4]

© 2021 Isaza N et al. *JAMA Network Open.*

III. Additional Results

a. Sensitivity of the ICER to Changes in Selected Variables

The ICER in our model was most sensitive to the variation in the annual cost of dapagliflozin cost, the effect of dapagliflozin on the risk of death in both patients without and with diabetes, and the incidence rate of diabetes among individuals without diabetes at baseline. This is depicted in the tornado plot included in Figure2, Panel B in the main manuscript.

b. Sensitivity Analysis of Null- or Negative Effect of Dapagliflozin on All-Cause Mortality

Assuming that dapagliflozin had a deleterious effect on all-cause mortality in patients without diabetes at baseline (HR=1.12, equal to the upper bound of the confidence interval in the published stratified analysis) made the use of dapagliflozin economically less attractive (with the ICER increasing to $117,200 per QALY gained). Similarly, assuming that dapagliflozin had a smaller effect in all-cause mortality in patients with diabetes (HR=0.97, equal to the upper bound of the confidence interval in the published stratified analysis) increased its ICER to $89,000 per QALY gained.

c. Sensitivity Analysis Assuming No Impact of Dapagliflozin on the Risk of Incident Diabetes

If dapagliflozin were assumed to have no effect on the risk of incident diabetes, the ICER for dapagliflozin and GDMT compared with GDMT alone in patients without diabetes at baseline increased from $68,300 per QALY gained to $73,500 per QALY gained.

© 2021 Isaza N et al. *JAMA Network Open.*

d. Sensitivity Analysis of Declining Effectiveness of Dapagliflozin

eAppendix Table 5 shows the results of a sensitivity analysis in which we assumed that the effectiveness of dapagliflozin on all-cause mortality would wean linearly for 5 years after trial completion. In this context, the ICER for dapagliflozin and GDMT compared with GDMT alone increased from $68,300 per QALY gained to $89,300 per QALY gained.

e. Sensitivity Analysis of Alternative Survival

eAppendix Table 6 shows the results for a sensitivity analysis in which we used an alternative survival model that used the pooled control arms of the PARADIGM-HF, EMPHASIS-HF and DAPA-HF trials.[3,17-19] In this context, the effectiveness of dapagliflozin increased as seen in the increase in the incremental life years and QALYs from 0.64 and 0.63 to 0.84 and 0.76 respectively. Although the improved survival led to an increase in the incremental healthcare costs from $42,800 to $54,900, the ICER for dapagliflozin and GDMT compared with GDMT alone decreased from $68,300 per QALY gained to $67,800 per QALY gained.

f. Additional Probabilistic Sensitivity Analysis Results

At a threshold of $100,000 per QALY, dapagliflozin, at an annual drug cost of $4,192, was cost-effective in 94% of 10,000 probabilistic simulations. This proportion declined to 72% simulations at a cost of $6,188 (wholesale acquisition price) but increased to 100%

© 2021 Isaza N et al. *JAMA Network Open.*

of simulations with a price of $953 (a heavily discounted price available in some US markets).

g. Sensitivity Analysis of Drug Discontinuation

In a sensitivity analysis evaluating the effect of drug discontinuation, assuming a monthly probability of discontinuing dapagliflozin of 0.27% for the first 2 years (to replicate the 4.7% discontinuation observed in the DAPA-HF trial) altered the ICER by less than 1% compared with the base case.

h. Acceptability curves

Acceptability curves (Figure 2) represent the proportion of 10,000 probabilistic simulations in which a strategy is cost-effective at varying thresholds. At cost-effectiveness thresholds of $50,000, $100,000, and $150,000 per quality-adjusted life year (QALY) gained, adding dapagliflozin to GDMT is projected to be cost-effective compared with GDMT alone in 0.05%, 94%, and 99% of the simulations respectively (Panel A). In subgroup analyses, similar results were obtained in patients without (Panel B) and with (Panel C) diabetes at baseline.

© 2021 Isaza N et al. *JAMA Network Open.*

**eAppendix Table 1. Estimated Cost of an Urgent Heart Failure Visit**			
Item	Charges, 2020 USD	Visits that received the service, %	Cost after adjustment for cost-center-specific charge-to-payment ratio and weighted for use, 2020 USD
Complete blood count	51	100	19.89
Serum Calcium	38	100	14.82
Serum Sodium	29	100	11.31
Serum Potassium	25	100	9.75
Serum Magnesium	81	100	31.59
Serum Chloride	25	100	9.75
Serum Bicarbonate	25	100	9.75
Blood Urea Nitrogen	25	100	9.75
Serum Creatinine	26	100	10.14
Brain Natriuretic Peptide	168	100	65.52
Electrocardiogram	143	100	55.77
Professional charges	368	100	143.52
Venipuncture	18	100	7.02
Transthoracic echocardiogram	1496	20	116.69
Lasix bolus	22	100	8.58
Lasix infusion	807	80	251.78
Chest X-ray	158	50	30.81
**Total**	**3505**	**-**	**806.44**

© 2021 Isaza N et al. *JAMA Network Open.*

**eAppendix Table 2. Mapping Kansas City Cardiomyopathy Questionnaire overall summary score to health-related quality-of-life weights.**	
	Point Estimates
a (intercept)	0.428986
B (slope)	0.005228

Model performance: $R^2 = 0.52$.[11] See section G (Quality-of-Life Parameters in the eAppendix for additional details.

© 2021 Isaza N et al. *JAMA Network Open.*

**eAppendix Table 3. Improvement in Quality-of-Life with Dapagliflozin Therapy Stratified by Baseline Diabetes Status – In-trial Observations**				
	No diabetes at baseline		Diabetes at baseline	
	GDMT	GDMT + Dapagliflozin	GDMT	GDMT + Dapagliflozin
Baseline	0.802	0.801	0.767	0.766
4m	0.826	0.835	0.791	0.800
8m	0.824	0.836	0.789	0.801

Abbreviations**:** GDMT = guideline-directed medical therapy

© 2021 Isaza N et al. *JAMA Network Open.*

eAppendix Table 4 Model Calibration

Calibration Measure	**Model Output**	**DAPA-HF Results (95% CI where available)**	**Difference in point estimates**
% Death from any cause at 18th months			
GDMT (Control)			
All patients	13%	13.1	-0.1%
No diabetes	11.3%	11.2%	+0.1%
Diabetes	15.4%	15.5%	-0.1%
Dapagliflozin (Intervention)			
All patients	11%	11.2%	-0.2%
No diabetes	10.0%	9.9%	+0.1%
Diabetes	12.2%	12.7%	-0.5%
Effect size*			
All patients	0.85	0.83 (0.71–0.97)	+0.02
No diabetes	0.88	0.88 (0.70 - 1.12)	0
Diabetes	0.79	0.78 (0.63 - 0.97)	+0.01
HF hospitalizations per patient-year			
GDMT (Control)			
All patients	0.1	0.1	0
No diabetes	0.09	0.08	+0.01
Diabetes	0.12	0.12	0
Dapagliflozin (Intervention)			
All patients	0.07	0.07	0
No diabetes	0.06	0.05	+0.01
Diabetes	0.09	0.09	0
Effect size*			
All patients	0.70	0.70 (0.59 to 0.83)	0
No diabetes	0.64	0.63 (0.48 - 0.81)	+0.01
Diabetes	0.76	0.76 (0.61 - 0.95)	0

Abbreviations: DAPA-HF = Dapagliflozin in Patients with Heart Failure and Reduced Ejection Fraction, CI = confidence interval, GDMT = guideline-directed medical therapy, HR = hazard ratio. *Effect size reported as the rate ratio observed in the model compared with the hazard ratio reported in the DAPA-HF trial, at 18.2 months (the median follow-up in the DAPA-HF trial).

© 2021 Isaza N et al. *JAMA Network Open.*

eAppendix Table 5. Sensitivity Analysis Assuming Declining Effectiveness of Dapagliflozin Beyond Trial Duration. Results of a sensitivity analysis in which we assumed that the effectiveness of dapagliflozin on all-cause mortality would wean linearly for 5 years after trial completion.

	All patients			No diabetes			Diabetes	
	GDMT	Dapagliflozin		GDMT	Dapagliflozin		GDMT	Dapagliflozin
**Health Outcomes**								
Survival, life years (undiscounted)	6.82(6.77-6.86)	7.27(7.21-7.32)		7.60(7.52-7.68)	8.01(7.90-8.10)		5.73(5.38-6.13)	6.24(5.88-6.63)
Survival, life years (discounted)	5.91(5.88-5.94)	6.28(6.23-6.31)		6.52(6.46-6.57)	6.84(6.76-6.90)		5.07(4.79-5.39)	5.49(5.21-5.80)
Incremental life years (discounted)	Comparator	0.37(0.33-0.40)		Comparator	0.32(0.25-0.38)		Comparator	0.42(0.42-0.43)
QALYs (discounted)	4.73(4.69-4.76)	5.09(5.05-5.13)		5.28(5.23-5.33)	5.63(5.56-5.69)		3.96(3.94-3.98)	4.35(4.35-4.36)
Incremental QALYs (discounted)	Comparator	0.36(0.33-0.40)		Comparator	0.35(0.29-0.40)		Comparator	0.40(0.38-0.41)
evLYG	Comparator	0.73(0.66-0.79)		Comparator	0.48(0.46-0.50)		Comparator	0.82(0.80-0.83)
**Direct Healthcare costs**								
Lifetime Healthcare Costs, 2020 USD (discounted)	150,609(131,290-172,215)	183,280(162,756-205,940)		149,000(142,600-174,700)	181,000(156,000-209,000)		152,900(126,000-183,400)	186,700(157,700-219,700)
Spending on dapagliflozin	-	26,313(26,123-26,465)		-	28,700(28,300-28,900)		-	23,000(21,900-24,300)
Spending on HF hospitalizations	6,971(5,158-9, 193)	5,129(3,660-7,011)		6,700(4,900-8,800)	4,400(3,100-6,200)		7,400(5,500-9,800)	6,100(4,200-8,500)
Incremental health costs, 2020 USD (discounted)	Comparator	32,700(31,500-33,725)		Comparator	32,000(13,400-34,300)		Comparator	33,800(31,700-36,300)
**ICER, $ per life-year gained**	Comparator	90,000(74,700 108,800)		Comparator	99,700(69,500-136,600)		Comparator	79,800($68,200-93,500)
**ICER, $ per QALY gained**	Comparator	89,300(80,100-99,500)		Comparator	92,100(66,400-124,000)		Comparator	85,900($72,900-102,000)
**ICER, $ per evLYG**	Comparator	44,700(40,500-49,700)		Comparator	69,800(66,500-67,600)		Comparator	43,700(40,900-41,800)

Abbreviations: GDMT = guideline-directed medical therapy, QALY = quality-adjusted life year, HF = heart failure, USD = United States dollars, - ICER = incremental cost-effectiveness ratio, evLYG = equal value of life years gained.
Numbers may not add up due to rounding.

© 2021 Isaza N et al. *JAMA Network Open.*

eAppendix Table 6. Sensitivity Analysis Using Alterative Survival Models in the Control Arm. In this sensitivity analyses, we modeled a more optimistic estimate of long-term survival based on a recently published analysis that pooled trial-level data from the control arms of 3 contemporary trials of HFrEF (3,17-19). Patients in the control arm of the studies were assumed to receive at least and angiotensin-converting–enzyme inhibitor, or an angiotensin-receptor blocker, or and a beta-blocker at the recommended or maximal tolerated dose.

	All patients			No diabetes			Diabetes	
	GDMT	Dapagliflozin		GDMT	Dapagliflozin		GDMT	Dapagliflozin
**Health Outcomes**								
Survival, life years (undiscounted)	8.12(8.06-8.18)	9.27(8.51-10.01)		9.07(8.97-9.16)	10.07(9.07-11.01)		6.81(6.80-6.82)	8.15(7.16-9.14)
Survival, life years (discounted)	6.82(6.78-6.86)	7.66(7.10-8.20)		7.53(7.45-7.59)	8.24(7.51-8.92)		5.84(5.84-5.85)	6.85(6.11-7.57)
Incremental life years (discounted)	Comparator	0.84(0.27-1.37)		Comparator	0.71(0.06-1.33)		Comparator	1.01(0.26-1.73)
QALYs (discounted)	5.44(5.40-5.48)	6.20(5.75-6.63)		6.08(6.01-6.14)	6.76(6.16-7.31)		4.66(4.54-4.58)	5.41(4.83-5.98)
Incremental QALYs (discounted)	Comparator	0.76(0.30-1.18)		Comparator	0.68(0.09-1.22)		Comparator	0.75(0.28-1.42)
evLYG	Comparator	0.91(0.35-1.45)		Comparator	0.81(0.07-1.47)		Comparator	1.08(0.33-1.79)
**Direct Healthcare costs**								
Lifetime Healthcare Costs, 2020 USD (discounted)	170,500(152,400-199,700)	225,400(195,800-258,300)		174,300(148,200-203,500)	221,200(186,400-260,700)		175,400(144,600-210,400)	231,100(188,300-280,300)
Spending on dapagliflozin	-	32,100(29,080-34,400)		-	46,900(38,200-57,200)		-	55,700(43,700-69,900)
Spending on HF hospitalizations	8,100(5,900-10,700)	6,400(4,500-8,700)		7,800(5,700-10,300)	5,500(3,700-7,700)		8,500(6,300-11,300)	7,600(5,200-10,700)
Incremental health costs, 2020 USD (discounted)	Comparator	54,900(47,200-64,800)		Comparator	46,900(40,300-55,300)		Comparator	55,700(47,900-65,700)
**ICER, $ per life-year gained**	Comparator	61,100 (45,100-Dominated)		Comparator	66,200(42,700-Dominated)		Comparator	56,100($38,800-234,300)
**ICER, $ per QALY gained**	Comparator	67,800(49,300-507,561)		Comparator	69,600(47,800-Dominated)		Comparator	65,700($46,700-182,600)
**ICER, $ per evLYG**	Comparator	55,200(44,900-95,700)		Comparator	58,100(43,000-281,100)		Comparator	52,200(42,300-94,300)

Abbreviations: GDMT = guideline-directed medical therapy, QALY = quality-adjusted life year, HF = heart failure, USD = United States dollars, - ICER = incremental cost-effectiveness ratio, evLYG = equal value of life years gained.
Numbers may not add up due to rounding.

© 2021 Isaza N et al. *JAMA Network Open.*

eAppendix Figure 1. Survival Model and Extrapolation Beyond Trial Duration. In trial survival stratified by diabetes status at baseline (solid orange and blue lines). Survival for 66-year-old in the general population based on 207 US Life Tables (solid black line). Extrapolation of survival in the base-case analysis (Dashed lines). Sensitivity analysis based on observations by Vaduganathan et. al.[17] See text for additional details.

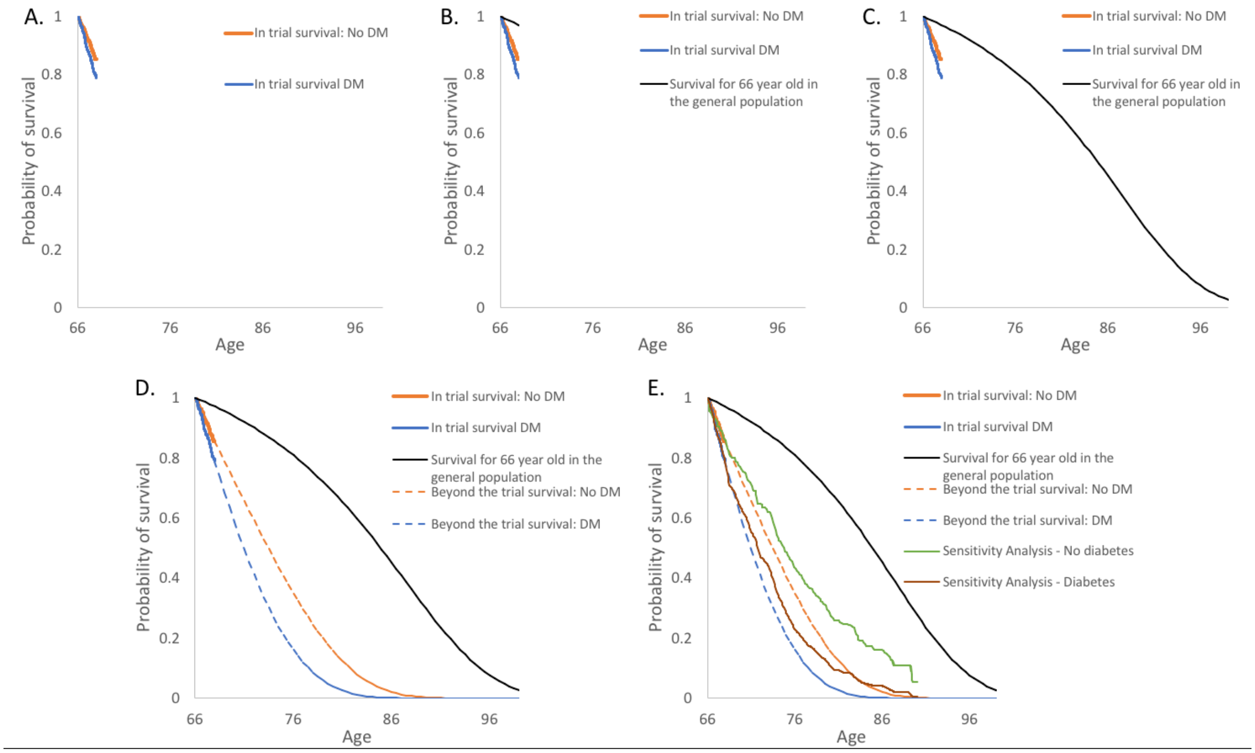

© 2021 Isaza N et al. *JAMA Network Open.*

eAppendix Figure 2. Acceptability curves.

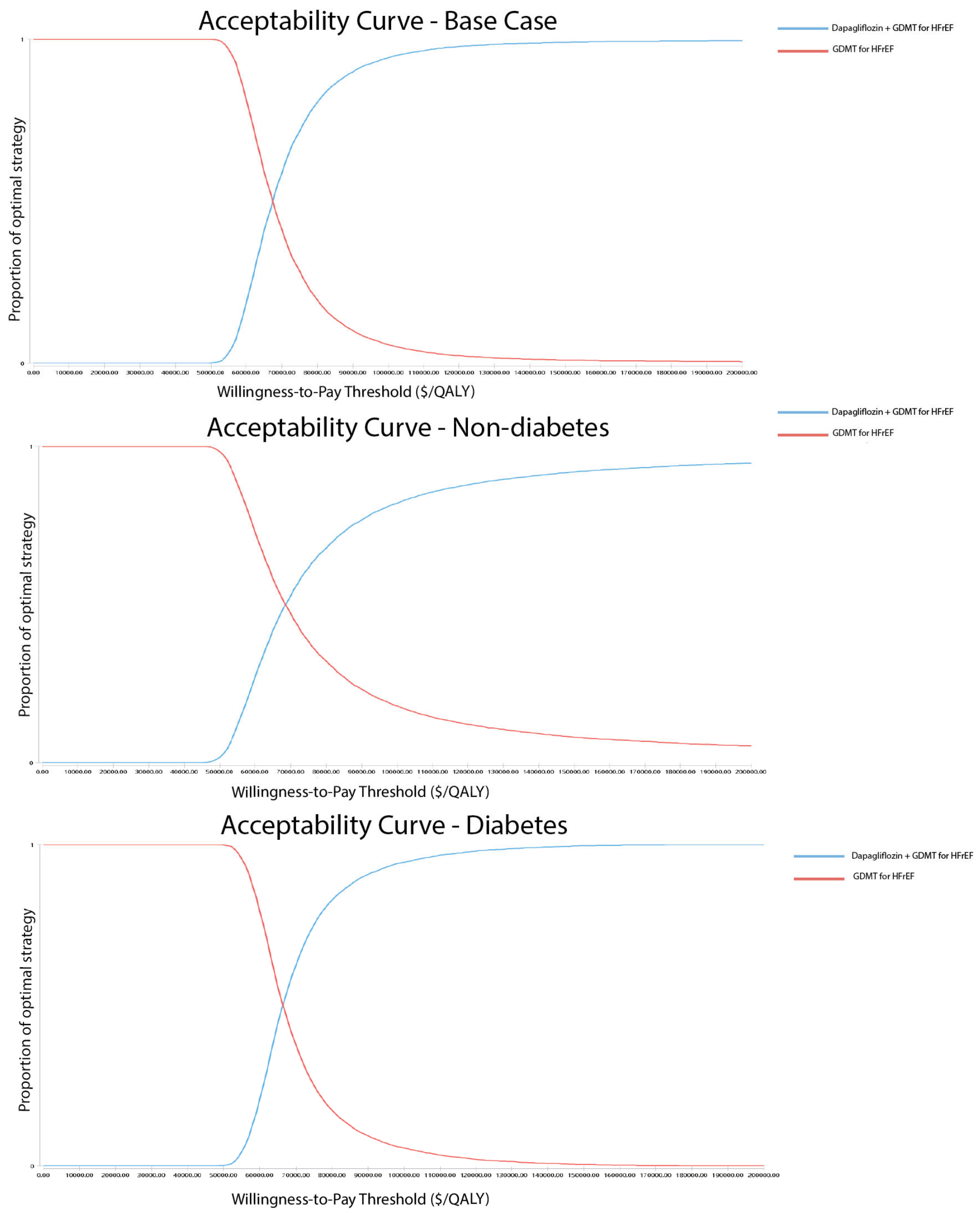

© 2021 Isaza N et al. *JAMA Network Open.*

eReferences

1. Yancy CW, Jessup M, Bozkurt B, et al. 2017 ACC/AHA/HFSA Focused Update of the 2013 ACCF/AHA Guideline for the Management of Heart Failure: A Report of the American College of Cardiology/American Heart Association Task Force on Clinical Practice Guidelines and the Heart Failure Society of America. *Circulation.* 2017;136(6):e137-e161.
2. Ponikowski P, Voors AA, Anker SD, et al. 2016 ESC Guidelines for the diagnosis and treatment of acute and chronic heart failure: The Task Force for the diagnosis and treatment of acute and chronic heart failure of the European Society of Cardiology (ESC)Developed with the special contribution of the Heart Failure Association (HFA) of the ESC. *Eur Heart J.* 2016;37(27):2129-2200.
3. McMurray JJV, Solomon SD, Inzucchi SE, et al. Dapagliflozin in Patients with Heart Failure and Reduced Ejection Fraction. *N Engl J Med.* 2019;381(21):1995-2008.
4. Petrie MC, Verma S, Docherty KF, et al. Effect of Dapagliflozin on Worsening Heart Failure and Cardiovascular Death in Patients With Heart Failure With and Without Diabetes. *JAMA.* 2020;323(14):1353-1368.
5. Healthcare Costs and Utilization Project. Statistics on Hospital Stays. https://hcupnet.ahrq.gov/ - setup. Accessed May 2020.
6. Peterson C, Xu L, Florence C, Grosse SD, Annest JL. Professional Fee Ratios for US Hospital Discharge Data. *Med Care.* 2015;53(10):840-849.
7. Wadhera RK, Joynt Maddox KE, Wasfy JH, Haneuse S, Shen C, Yeh RW. Association of the Hospital Readmissions Reduction Program With Mortality Among Medicare Beneficiaries Hospitalized for Heart Failure, Acute Myocardial Infarction, and Pneumonia. *JAMA.* 2018;320(24):2542-2552.
8. Farxiga approved in the US to reduce the risk of hospitalisation for heart failure in patients with type-2 diabetes [press release]. AstraZeneca21 October, 2019.
9. US Food and Drug Administration . Farxiga (dapagliflozin) [package insert]. U.S. Food and Drug Administration website. https://www.accessdata.fda.gov/drugsatfda_docs/label/2020/202293s020lbl.pdf. Revised May 2020. Accessed May 21, 2020.
10. Kosiborod MN, Jhund PS, Docherty KF, et al. Effects of Dapagliflozin on Symptoms, Function, and Quality of Life in Patients With Heart Failure and Reduced Ejection Fraction: Results From the DAPA-HF Trial. *Circulation.* 2020;141(2):90-99.
11. Kazi DS, Bellows BK, Baron SJ, et al. Cost-Effectiveness of Tafamidis Therapy for Transthyretin Amyloid Cardiomyopathy. *Circulation.* 2020;141(15):1214-1224.
12. Sullivan PW, Ghushchyan V. Preference-Based EQ-5D index scores for chronic conditions in the United States. *Med Decis Making.* 2006;26(4):410-420.
13. Jaagosild P, Dawson NV, Thomas C, et al. Outcomes of acute exacerbation of severe congestive heart failure: quality of life, resource use, and survival. SUPPORT Investigators. The Study to Understand Prognosis and Preferences for Outcomes and Risks of Treatments. *Arch Intern Med.* 1998;158(10):1081-1089.
14. Inzucchi SE, Docherty K, Kober L, et al. Effect of Dapagliflozin on the Incidence of Diabetes: A Prespecified Exploratory Analysis from DAPA-HF. *Diabetes.* 2020;69(Supplement 1):271-OR.

© 2021 Isaza N et al. *JAMA Network Open.*

15. Krumholz HM. Post-hospital syndrome--an acquired, transient condition of generalized risk. *N Engl J Med.* 2013;368(2):100-102.
16. National Center for Health Statistics. United States Life Tables, 2017. https://www.cdc.gov/nchs/products/life_tables.htm. Published 2019. Accessed2020.
17. Vaduganathan M, Claggett BL, Jhund PS, et al. Estimating lifetime benefits of comprehensive disease-modifying pharmacological therapies in patients with heart failure with reduced ejection fraction: a comparative analysis of three randomised controlled trials. *Lancet.* 2020;396(10244):121-128.
18. McMurray JJ, Packer M, Desai AS, et al. Angiotensin-neprilysin inhibition versus enalapril in heart failure. *N Engl J Med.* 2014;371(11):993-1004.
19. Zannad F, McMurray JJ, Krum H, et al. Eplerenone in patients with systolic heart failure and mild symptoms. *N Engl J Med.* 2011;364(1):11-21.
20. Neumann PJ, Ganiats TG, Russell LB, Sanders GD, Siegel JE, Oxford University P. *Cost-effectiveness in health and medicine. Chapter 8, Estimating Costs and Valuations of Non-Health Benefits in Cost Effectiveness Analysis.* Second Edition ed. New York: Oxford University Press; 2017.
21. Office of Procurement, Acquisition and Logistics. Pharmaceutical Prices. https://www.va.gov/opal/nac/fss/pharmPrices.asp. Accessed August 2020.
22. Sanders GD, Neumann PJ, Basu A, et al. Recommendations for Conduct, Methodological Practices, and Reporting of Cost-effectiveness Analyses: Second Panel on Cost-Effectiveness in Health and Medicine. *JAMA.* 2016;316(10):1093-1103.
23. SSR Health. US Brand Rx Net Price. http://www.ssrhealth.com/research-archive/. Accessed August 2020.
24. SSR Health. US Brand Rx WAC / Net Price. http://www.ssrhealth.com/research-archive/. Accessed August 2020.
25. Centers for Medicare and Medicaid Services. Medicare Provider Utilization and Payment Data. https://www.cms.gov/Research-Statistics-Data-and-Systems/Statistics-Trends-and-Reports/Medicare-Provider-Charge-Data/Physician-and-Other-Supplier. Published 2017. Accessed2020.
26. Agency for Healthcare Research and Quality. Medical Expenditure Panel Survey. https://meps.ahrq.gov/mepsweb/. Accessed May, 2020.
27. Spertus JA, Jones PG, Sandhu AT, Arnold SV. Interpreting the Kansas City Cardiomyopathy Questionnaire in Clinical Trials and Clinical Care: JACC State-of-the-Art Review. *J Am Coll Cardiol.* 2020;76(20):2379-2390.
28. Green CP, Porter CB, Bresnahan DR, Spertus JA. Development and evaluation of the Kansas City Cardiomyopathy Questionnaire: a new health status measure for heart failure. *J Am Coll Cardiol.* 2000;35(5):1245-1255.
29. Spertus J, Peterson E, Conard MW, et al. Monitoring clinical changes in patients with heart failure: a comparison of methods. *Am Heart J.* 2005;150(4):707-715.
30. Nord E, Pinto JL, Richardson J, Menzel P, Ubel P. Incorporating societal concerns for fairness in numerical valuations of health programmes. *Health Econ.* 1999;8(1):25-39.

© 2021 Isaza N et al. *JAMA Network Open.*
